# The HIV-1 epidemic in Bolivia is dominated by subtype B and CRF12_BF "family" strains

**DOI:** 10.1186/1743-422X-9-19

**Published:** 2012-01-16

**Authors:** Monick L Guimarães, Ketty G Velarde-Dunois, David Segurondo, Mariza G Morgado

**Affiliations:** 1 Laboratório de AIDS & Imunologia Molecular, Instituto Oswaldo Cruz, Rio de Janeiro, RJ, Brazil; 2 Policía Nacional, La Paz, Bolivia; 3 Programa Departamental de ITS, VIH/SIDA, Ministerio de la Salud, La Paz, Bolivia; 4 Laboratório de AIDS & Imunologia Molecular, IOC/FIOCRUZ, Av. Brasil 4365, Leonidas Deane, room #413, Rio de Janeiro 21040-360, Brazil

**Keywords:** HIV-1, Bolivia, Subtypes, CRF

## Abstract

**Background:**

Molecular epidemiological studies of HIV-1 in South America have revealed the occurrence of subtypes B, F1 and BF1 recombinants. Even so, little information concerning the HIV-1 molecular epidemiology in Bolivia is available. In this study we performed phylogenetic analyses from samples collected in Bolivia at two different points in time over a 10 year span. We analyzed these samples to estimate the trends in the HIV subtype and recombinant forms over time.

**Materials and methods:**

Fifty one HIV-1 positive samples were collected in Bolivia over two distinct periods (1996 and 2005). These samples were genetically characterized based on partial *pol protease/reverse transcriptase (pr/rt) *and *env *regions. Alignment and neighbor-joining (NJ) phylogenetic analyses were established from partial *env *(n = 37) and all *pol *sequences using Mega 4. The remaining 14 *env *sequences from 1996 were previously characterized based on HMA-*env *(Heteroduplex mobility assay). The Simplot v.3.5.1 program was used to verify intragenic recombination, and SplitsTree 4.0 was employed to confirm the phylogenetic relationship of the BF1 recombinant samples.

**Results:**

Phylogenetic analysis of both *env *and *pol *regions confirmed the predominance of "pure" subtype B (72.5%) samples circulating in Bolivia and revealed a high prevalence of BF1 genotypes (27.5%). Eleven out of 14 BF1 recombinants displayed a mosaic structure identical or similar to that described for the CRF12_BF variant, one sample was classified as CRF17_BF, and two others were F1*pol*/B*env*. No "pure" HIV-1 subtype F1 or B" variant of subtype B was detected in the present study. Of note, samples characterized as CRF12_BF-related were depicted only in 2005.

**Conclusion:**

HIV-1 genetic diversity in Bolivia is mostly driven by subtype B followed by BF1 recombinant strains from the CRF12_BF "family". No significant temporal changes were detected between the mid-1990s and the mid-2000s for subtype B (76.2% vs 70.0%) or BF1 recombinant (23.8% vs 30.0%) samples from Bolivia.

## Background

Latin America contains the third highest estimated number of people living with HIV in the world [1]. The HIV epidemic in countries from the Southern Cone is characterized by the co-circulation of HIV-1 subtypes B, F1, and BF1 recombinant forms [2-4]. Despite such overall similarity, the mosaic patterns of the HIV-1 BF1 Circulanting Recombinant Forms (CRFs_BF) and Unique Recombinant Forms (URFs_BF) display some important differences among countries.

The HIV-1 BF1 epidemics in Argentina [5-9], Chile [10], Paraguay [11] and Uruguay [12] are dominated by the CRF12_BF and URFs_BF sharing CRF12-related structures, in other words sharing some of CRF12_BF breakpoints. In addition, new local CRFs_BF with a CRF12-related structure have been identified within each country, including CRF17_BF in Argentina and Paraguay [9,11], CRF38_BF in Uruguay [12], and CRF44_BF in Chile [13]. By contrast, the HIV-1 BF1 Brazilian epidemic is driven mainly by a myriad of different URFs_BF and at least five distinct CRFs_BF [14-19], unrelated to CRF12_BF, which usually show no common mosaic recombinant profile among one another. Another important difference is that some "pure" full-length subtype F1 genomes have been described in Brazil [20], while the frequency of such full-length subtype F1 viruses or even non-recombinant subtype F1 *pol *sequences is almost absent in other South American countries [8,12,21,22].

Bolivia is a landlocked country in central South America, bordered by Brazil to the north and east, Paraguay and Argentina to the south, and Chile and Peru to the west. The total number of HIV/AIDS cases reported in Bolivia was 5,184 from 1984 through March 2010 [23], with an estimated country-wide underreporting rate of over 70% [24]. UNAIDS estimates that approximately 12,000 people in Bolivia were living with HIV in 2009, and of those cases, 500 to 1500 were new infections [1]. These estimates demonstrate that the Bolivian epidemic is still potentially growing. Very scarce data on HIV epidemiology in Bolivia are available, with only two major previous studies conducted in the 1990s, which revealed the occurrence of subtypes B, F1 and BF1 recombinant forms [22,25], similar to what has been described for other countries from the Southern Cone [2-13]. In these previous studies, however, most samples were genetically characterized on the basis of HMA *env *analysis, and very few (*n *= 14) HIV-1 nucleotide sequences from Bolivian seropositive patients are available at the Los Alamos HIV database, from those only three were full length genomes that reveal the circulation of CRF12_BF [5] and CRF17_BF.

The objective of the present study was to assess the trends in the HIV molecular epidemiology in Bolivia based on the phylogenetic analysis of HIV-1 *pol *and *env *sequences at two different time points (1996 and 2005).

## Results

**Figure 1:**
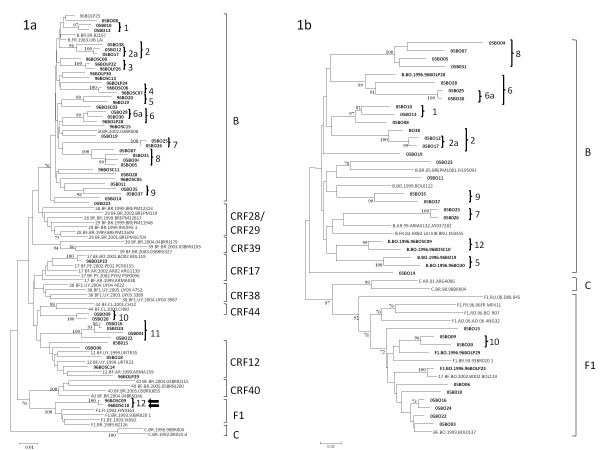
**Phylogenetic analysis of 51 HIV-1 *pol *(a) and 37 *env *(b) samples from Bolivia**. Bootstrap values ≥70% supporting nodes are shown on the left of each node and were estimated using the neighbor-joining with Tamura-Nei substitution model. The scale bar indicates 10% nucleotide sequence divergence. HIV-1 samples analyzed in the present study were in bold format. Arrows indicate those samples with intergenic recombination. Highly supported (> 95%) monophyletic clusters are highlighted and numbered.

**Figure 2:**
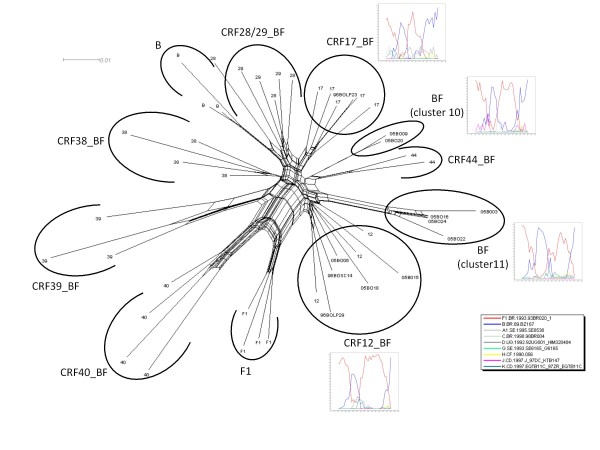
**SplitsTree analysis of the HIV-1 Bolivian BF1 *pol *regions**. The splits graph was constructed using NeighborNet methodology with pairwise distance input, which was estimated by F84 distance. The scale bar indicates 10% nucleotide sequence divergence. Reference sequences were indicated by subtype letter or CRF number. The alignment was prepared with HIV-1 group M subtypes B and F1 and CRF_BFs from South America. Four clusters containing studied samples were detected and are indicated with a circle. Representative bootscanning plots were generated for each cluster. Bootscan analysis was performed on a sliding window of 200 nt of the query sequences moving along an alignment of reference sequences by increments of 20 nt. Reference samples used for these analyses are discriminated in the figure legend.

In order to assess the trends of the HIV-1 molecular epidemiology in Bolivia, 62 samples were collected at two points in time, 1996 (30 samples) and 2005 (32 samples), and submitted to amplification and sequencing of both the *pol *(*pr/rt) *and *env *genomic regions. Eleven samples (nine from 1996 and two from 2005) failed to be amplified or sequenced from the proviral DNA in one or both genomic regions and were excluded from the study. The remaining 51 samples showed a predominance of the B subtype (72.5%) and BF1 recombinants (27.5%) (Figure 1). Of note, no pure subtype F1 genomes (F1*pol*/F1*env*) were detected. Fourteen highly supported (> 95%) monophyletic clusters of at least two sequences could be detected in the *pol *(Figure 1a) and 11 in *env *region (Figure 2b). Some clusters were composed of sequences retrieved from individuals with a known direct epidemiological link, such as sexual partners (clusters 2a, 5 and 6a), and a mother/child pair (cluster 7). No apparent direct epidemiological relationships were detected for the remaining 10 clusters (1, 2, 3, 4, 6, 8, 9, 10, 11, 12). Among the 12 monophyletic clusters, nine displayed a B*pol*/B*env *profile (1-9), two were BF1*pol*/F1*env *(10, 11), and one was F1*pol*/B*env *(12) (Figure 1).

The 14 BF1 recombinant samples identified in this study displayed the following recombination profiles: BF1*pol*/F1*env *(*n *= 11), BF1*pol*/B*env *(*n *= 1) and F1*pol*/B*env *(*n *= 2) (Figure 1). A more detailed evaluation of the 12 BF1 *pol *recombinant samples was performed using bootscan and SplitsTree analyses. From these, five samples displayed a recombination profile in *pr/rt *identical to the CRF12_BF, being classified as CRF12_BF-like (96BOLP29, 96BOSC14, 05BO06, 05BO15, 05BO18), and one sample exhibited a CRF17_BF-like mosaic pattern (96BOLP23) (Figure 2).

**Figure 3:**
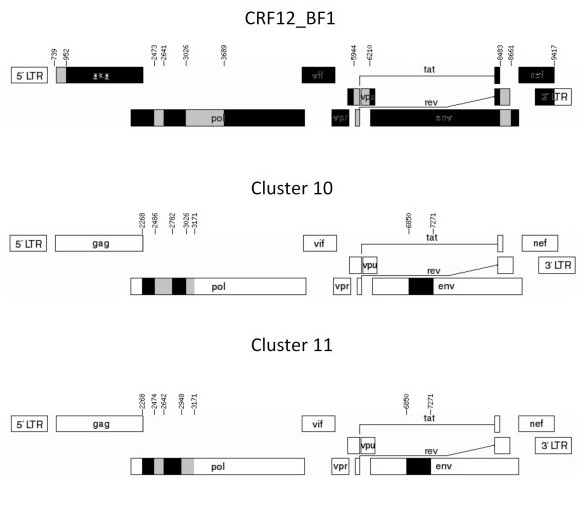
**Schematic representation of the BF1 mosaic structure from clusters 10, 11 and CRF12**. Both genomic structures were drawn by using the Recombinant Draw Toll available in the Los Alamos homepage (http://www.hiv.lanl.gov/content/hiv-db/DRAW_CRF/recom_mapper.html).

No epidemiological data were available to confirm the proposed close genetic relationship between the CRF17_BF (BO02.BO119) strain available in the Los Alamos Sequence Database and the BF recombinant genome described in the present study. The remaining six BF1 *pol *samples segregate in two highly supported monophyletic clusters and exhibited two different CRF12_BF-related (F1/B/F1/B) mosaic profiles (Figure 2). In the first cluster (cluster 10), which is composed of samples 05BO09 and 05BO20, the first (F1/B) and second (B/F1) recombination breakpoints at *pol *coincide with those observed in the CRF12_BF, while the third one (F1/B) is displaced downstream. In the second cluster (cluster 11), which is composed of samples 05BO03, 05BO16, 05BO22 and 05BO24, only the third (F1/B) recombination breakpoint at *pol *coincides with those observed in the CRF12_BF, while the first (F1/B) and the second (B/F1) recombination breakpoints at *pol *were displaced upstream in comparison to those detected in CRF12_BF (Figure 3). None of the CRFs_BF *pol *mosaic patterns described in Brazil (CRF28, CRF29, CRF39, and CRF40), Uruguay (CRF38), or Chile (CRF44) was observed for the HIV-1 samples obtained from Bolivia.

Table 1 summarizes the subtype distribution and recombination profiles of the 51 HIV-1 samples from Bolivia according to the time period. No significant temporal change in the HIV-1 subtype distribution was observed in Bolivia between 1996 and 2005. During this period the prevalence of subtype B decreased from 76.2% to 70%, while the prevalence of BF1 recombinants increased from 23.8% to 30%.

**Table 1:** HIV-1 genetic forms identified in the 51 HIV-1 samples from Bolivia in two distinct time periods (1996 and 2005)

Genotype	1996 (n = 21)	%	2005 (n = 30)	%
B *pol*/B *env*	16	76.2	21	70
BF1 recombinants	5	23.8	9	30
F1 *pol*/B *env*	2	9.5	0	0
CRF12-like *pol*/B *env*	1	4.8	0	0
CRF12-like *pol*/F1 *env*	1	4.8	3	10
CRF12-related *pol*/F1 *env*	0	0	6	20
CRF17-like *pol/F1 env*	1	4.8	0	0

*List of abbreviations*: *HIV-1 *Human immunodeficiency virus-1, *pol *Polymerase, *pr/rt *Protease/reverse transcriptase, *env *Envelope, *NJ *neighbor-joining, *CRF *Circulating recombinant forms, *URF *Unique recombinant forms, *AIDS *Acquired immune deficiency syndrome, *HMA *Hetereduplex mobility assay, *DNA *Deoxyribonucleic acid

## Discussion

The present study represents the most comprehensive analysis of the HIV-1 molecular diversity in Bolivia performed to date. From a total of 51 samples investigated in both *pol *(*pr/rt*) and *env *(C2-V3) regions, 72.5% of cases were classified as "pure" subtype B, and 27.5% were classified as BF1 recombinants. Eleven out of 14 BF1 recombinants displayed a mosaic structure identical or similar to that described for the CRF12_BF strain, and the remaining two BF1 recombinants displayed a F1(*pol*)/B(*env*) mosaic structure. Subtype F1 (*pol*)/F1 (*env*) strains were not detected in the present study.

The high frequency of BF1 recombinants with CRF12_BF-like or CRF12_BF-related structures and the absence of non-recombinant subtype F1 strains found in Bolivia reveal a scenario closely related to that found in Argentina, Chile, Paraguay and Uruguay [6-12], and different from that found in other neighboring countries, such as Brazil and Peru [17-19,22]. This pattern suggests a more intense HIV-1 influx into Bolivia from the southern border (Argentina and/or Paraguay) than from the eastern (Peru) or western/northern (Brazil) frontiers. It is interesting to note that none of the subtype B samples studied presented the typical Brazilian motif GWGR in the top of the v3 loop (B" variant of subtype B), which is highly (40%) represented in Brazil [26]. The reason for such potential unequal viral genetic influxes into Bolivia from neighboring countries remains unknown and deserves further investigation.

In addition to the widely disseminated CRF12_BF variant, other CRFs_BF variants with a CRF12_BF-related structure were also described as circulating in Argentina and Paraguay (CRF17_BF), Uruguay (CRF38_BF) and Chile (CRF44_BF) [9-13]. One sequence with a CRF17_BF-like recombinant structure was also detected in our study, confirming the circulation of this CRF in Bolivia since 1996. Of note, we also detected one highly supported cluster (cluster 11) of four BF1 sequences displaying the same CRF12_BF-related (*pol*)/F1 (*env*) mosaic profile, obtained from epidemiologically unlinked HIV-1 seropositive patients from Bolivia. Such a result may represent a new local Bolivian CRF_BF variant. Full-length genome analysis of these four BF1 sequences will be necessary to confirm this hypothesis.

It is interesting to note that all BF1 recombinants with a CRF12_BF-related structure detected in Bolivia displayed larger subtype B segments at *pol *than CRF12_BF. A similar finding was also observed in Argentina [5-9] and Chile [10]. The generation of CRF12_BF-related recombinants with larger subtype F1 fragments in those countries is unexpected because of the scarcity (or complete absence) of pure subtype F1 variants outside of Brazil. It has been proposed that the CRF12_BF strain and other CRFs_BF and URFs_BF variants with related structures circulating in South America, as well as other groups of related recombinants should be considered components of the same "family" of recombinants [3,27].

Since its emergence around the early 1980s, the CRF12_BF variant has displayed an initial exponential expansion in Argentina and Uruguay [28]. Such a fast rate of spread and the high prevalence (> 50%) of CRF12_BF and CRF12_BF-related mosaic forms in those countries could be interpreted as evidence of the high viral fitness and/or transmissibility of the CRF12 "family" of recombinants. However, much lower prevalence (< 25%) of CRF12_BF-like and CRF12_BF-related variants has been found in Bolivia [this study], Chile [10] and Paraguay [11]. Our results did not depict a significant increase in the proportion of those mosaic HIV-1 forms in Bolivia between 1996 and 2005, nevertheless all samples from 2005 were collected in only one city: La Paz. This finding suggests that the rate of expansion and the resulting prevalence of CRF12_BF "family" variants in South America are country-specific and might be dependent on non-virological factors. It has been also proposed that the prevalence of CRF12_BF and related BF1 strains is higher in countries with more extensive intravenous drug epidemics, possibly due to a founder effect and the rapid spread of HIV in these groups [22].

## Conclusion

In summary, our results demonstrate that HIV-1 genetic diversity in Bolivia is mostly driven by subtype B followed by BF1 recombinant strains from the CRF12_BF "family" and no significant temporal changes in the subtype distributions were detected between the mid-1990s and the mid-2000s.

## Materials and methods

### Study population

A set of 62 samples were collected in Bolivia in 1996 (30 samples) and 2005 (32 samples). Demographic, laboratory and clinical data of Bolivian subjects collected in 1996 were previously described [24]. Samples from 1996 were collected in three distinct Bolivia cities [La Paz (n = 9), Cochabamba (n = 5) and Santa Cruz (n = 16)]. The second cohort was composed exclusively of patients living in La Paz, and most of them were under antiretroviral therapy. All participants signed the informed consent form, and the study was approved by the Bolivian Ministry of Health.

### HIV-1 DNA amplification and sequencing

Genomic DNA was extracted from whole blood by the phenol/chloroform method from samples collected in 1996, as described [25]. For samples collected in 2005, the QIAamp DNA kit was used (Qiagen Inc., CA, U.S.A.) according to the manufacturer's protocol.

Amplification of HIV-1 *env *and *pol *regions was performed using an in-house nested PCR protocol method as described elsewhere [29]. Samples collected in 1996 were previously subtyped based on the HMA env-gp120 C2-V3 region and 11 of them were also sequenced and phylogenetically analyzed as described [25]. Partial protease/reverse transcriptase (*pr/rt*) sequences were generated for the samples from both periods, and new *env *sequences were obtained only from samples collected in 2005. No HIV-1 seropositive individuals from the first time period were undergoing antiretroviral therapy at the time of blood collection; however, more than a half of the individuals in the second group were being treated.

### HIV-1 subtype classification

HIV-1 subtype classification was performed by phylogenetic analysis with the neighbor-joining method using the Tamura-Nei model as implemented in Mega 4.0 [30]. Those samples that present divergent clustering in the phylogenetic analysis were investigated for recombination by bootscan analysis as implemented in the Simplot version 3.5.1 (http://www.med.jhu.edu/deptmed/sray/download/), using representative HIV-1 sequences from group M. Bootstrap values supporting branching with reference sequences were determined in neighbor-joining trees constructed using the Kimura 2-parameter model, based on 100 re-samplings, with a 200 nucleotide sliding window moving in steps of 20 bases. The SplitsTree program version 4 [31] was employed to confirm the phylogenetic relationship of the recombinant samples using the NeighborNet based on the pairwise distance estimated by F84 parameter model.

### Sequence data

GenBank accession numbers: JN710748-JN710798 (*env-gp120 *C2-V3 region), JN710799-JN710830 (*pr/rt*).

## Competing interests

The authors declare that they have no competing interests.

## Authors' contributions

M.L.G., K.G.V.D and M.G.M conceived and designed the study. D.S and K.G.V.D were responsible for patients' recruitment and sample collection in Bolivia. K.G.V.D, M.L.G. and M.G.M. performed the characterization of the samples from 1996 in the *env *region. M.L.G. conducted the characterization of the *pol *region from both studied periods. M.L.G performed the phylogenetic analyses. M.L.G. and M.G.M wrote the first draft and all authors contributed to the final version of the paper.
